# The Effects of Repetitive Transcranial Magnetic Stimulation on Anxiety in Patients With Moderate to Severe Traumatic Brain Injury: A *Post-hoc* Analysis of a Randomized Clinical Trial

**DOI:** 10.3389/fneur.2020.564940

**Published:** 2020-12-04

**Authors:** Priscila Aparecida Rodrigues, Ana Luiza Zaninotto, Hayden M. Ventresca, Iuri Santana Neville, Cintya Yukie Hayashi, Andre R. Brunoni, Vinicius Monteiro de Paula Guirado, Manoel Jacobsen Teixeira, Wellingson Silva Paiva

**Affiliations:** ^1^Department of Neurology, University of São Paulo, São Paulo, Brazil; ^2^Speech and Feeding Disorders Lab, Massachusetts General Hospital Institute of Health Professions (MGHIHP), Boston, MA, United States; ^3^Laboratory of Neurosciences (LIM-27), Department and Institute of Psychiatry, Faculdade de Medicina da Univerdade de São Paulo, Instituto Nacional de Biomarcadores em Neuropsiquiatria (INBioN), São Paulo, Brazil; ^4^Department of Internal Medicine, Faculdade de Medicina da Universidade de São Paulo & Hospital Universitário, Universidade de São Paulo, São Paulo, Brazil

**Keywords:** traumatic brain injury, anxiety disorder, executive function, depression, transcranial magnetic stimulation, neuropsychology

## Abstract

**Background:** Traumatic brain injury (TBI) is one of the leading causes of neuropsychiatric disorders in young adults. Repetitive Transcranial Magnetic Stimulation (rTMS) has been shown to improve psychiatric symptoms in other neurologic disorders, such as focal epilepsy, Parkinson's disease, and fibromyalgia. However, the efficacy of rTMS as a treatment for anxiety in persons with TBI has never been investigated. This exploratory *post-hoc* analyzes the effects of rTMS on anxiety, depression and executive function in participants with moderate to severe chronic TBI.

**Methods:** Thirty-six participants with moderate to severe TBI and anxiety symptoms were randomly assigned to an active or sham rTMS condition in a 1:1 ratio. A 10-session protocol was used with 10-Hz rTMS stimulation over the left dorsolateral prefrontal cortex (DLPFC) for 20 min each session, a total of 2,000 pulses were applied at each daily session (40 stimuli/train, 50 trains). Anxiety symptoms; depression and executive function were analyzed at baseline, after the last rTMS session, and 90 days post intervention.

**Results:** Twenty-seven participants completed the entire protocol and were included in the *post*-*hoc* analysis. Statistical analysis showed no interaction of group and time (*p* > 0.05) on anxiety scores. Both groups improved depressive and executive functions over time, without time and group interaction (*p*_*s*_ < 0.05). No adverse effects were reported in either intervention group.

**Conclusion:** rTMS did not improve anxiety symptoms following high frequency rTMS in persons with moderate to severe TBI.

**Clinical Trial Registration:** www.ClinicalTrials.gov, identifier: NCT02167971.

## Introduction

Moderate to severe Traumatic Brain Injury (TBI) is one of the greatest worldwide burden of diseases (1–3). Accidental injury, the main cause of TBI, is the leading cause of death among people under 45 years-old (4). Those who survive a TBI are often left with lifelong neurologic sequelae (4, 5). A recent Brazilian study calculated an annual incidence of 9.5 cases of severe TBI per 100,000 inhabitants (6) and ~69 million individuals suffer from TBI each year worldwide (7). TBIs are classified into four major groups according to the type of trauma, including skull fractures, focal lesions, diffuse lesions, and penetrating lesions (8). Diffuse lesions are common in moderate to severe TBI cases (9–11), and generally do not occur in a specific location of the brain or present macroscopic structural damage, instead involving substantial volume of widespread axonal disruption, particularly in the white matter (12, 13). Diffuse lesions usually occurs when the brain is violently shifted or rotated within the skull, which causes microscopic injuries to broad swaths of axons. Diffuse lesions affects about 40% of patients with severe TBI (8, 14) and is responsible for almost 1/3 of TBI-related deaths (15).

Anxiety is one of the major psychiatric disorders that can result from TBI (16). Studies have suggested that 40–60% of patients with moderate to severe TBI meet criteria for an anxiety disorder 1-year post-injury (16–18), in part due to the patients' inability to perform pre-injury activities, the uncertainty of recovery, and decrease in their quality of life (16). During the chronic phase of TBI, the proportion of anxiety symptoms and/or generalized anxiety disorder remains stable, and patients present with the same degree of severity (19, 20). Forty-six percent of the patients with moderate to severe TBI may present with an anxiety disorder during the course of their disease (18, 21).

Previous studies have shown that depressive symptoms are often concomitant with anxiety symptoms (22–25), with a comorbidity rate of up to 60% in cases of moderate to severe TBI (26, 27). Anxiety further impairs executive functioning skills such as impulse control, organization, and planning (16, 28). Previous studies indicate that anxiety symptoms decrease cognitive and functional outcomes in TBI (18, 26). The impact of anxiety on executive functioning is caused by neural disruptions in frontotemporal, prefrontal and limbic regions (29). Those same regions are most likely to be damaged in a moderate to severe TBI and are associated with psychiatric and behavioral impairment in this population (30).

Despite the high prevalence of anxiety disorder in persons with TBI, there is a shortage of treatments for these symptoms (31). Further, medication is not always effective, and its long-term use can cause adverse side effects (32). Non-invasive brain stimulation (NIBS) is a viable but underexplored treatment for anxiety in this population. NIBS has been demonstrated to modulate neural activity (33, 34) by engendering synaptic plasticity (33, 35, 36). Recent evidence suggests that NIBS is a viable alternative treatment for pathological synaptic states underlying a range of neurological, cognitive, and psychiatric disorders (37–40), including anxiety (41–47). Transcranial magnetic stimulation (TMS) is the NIBS modality most commonly used by clinicians, primarily as an intervention for treatment-resistant major depression (48). Although few studies have investigated the efficacy of the TMS in patients with TBI (49–51), existing literature demonstrates a link between TMS therapies, increased neuroplasticity, and improved cognition, particularly in regards to executive functions and mood (52). In psychiatric populations, repetitive TMS (rTMS) has been used to decrease anxiety symptoms in Generalized Anxiety Disorder (43–45), Panic Disorder (53, 54), Obsessive-compulsive disorders (55), Post-Traumatic Stress Disorder (56–58), Borderline (59), Major Depressive Disorder (60, 61), and Schizophrenia (62). However, there is no consensus on the optimal parameters of rTMS as a treatment for anxiety symptoms in persons with TBI. Previous studies have shown that high frequency (10–20 Hz) stimulation of the dorsolateral prefrontal cortex (DLPFC) promotes excitatory stimulation (43–46) and can improve anxiety symptoms in patients with neurological disorders (63). Despite initial concerns regarding the use of rTMS on patients with TBI, the safety of this modality has since been demonstrated in this population (64–66).

The present study is the first randomized clinical trial to investigate anxiety outcomes after 10 consecutive sessions of high-frequency rTMS in patients with chronic moderate to severe TBI. Due to the high incidence of diffuse lesions in moderate to severe TBI and the widespread non-specific brain lesion damage location (9, 67, 68), this is an excellent population to investigate the efficacy of rTMS intervention on anxiety symptoms. This study is the result of a *post-hoc* analysis of a clinical trial that evaluated the effects of rTMS on attention and executive functions (66). The current *post-hoc* analysis aims (1) to investigate the effect of 10 sessions of high frequency rTMS on anxiety symptoms in patients with moderate to severe TBI, (2) to analyze changes in depressive scores, and (3) executive function index after the rTMS between active and control rTMS. We followed the CONSORT statement to structure and write this paper.

## Methods

Patients were recruited from the Neurotrauma Outpatient Clinic of the Hospital das Clínicas of the Medical School of the University of São Paulo (HCFMUSP) from 2014 to 2016. Only patients who completed at least 8 out of 10 rTMS sessions were included in analysis. This research was approved by the Research Ethics Committee (CAPPESQ) of HCFMUSP under n°. 1.151.170.

### Participants and Setting

Demographic, medical history, and injury data were collected and verified through interviews and medical record by a registered nurse (CYH) or the neurosurgeon (ISN).

#### Inclusion Criteria

Included in this study patients aged 18–60 years; sustained a non-penetrating TBI at least 12 months before enrollment; stable medication regimen for at least 1 month before enrollment with no plans to change medications during the 90-day study window; anxiety symptoms higher than 41 points (STAI-State score >41) (69, 70); a clinically and radiologically [computer tomography (CT) scan or structural magnetic resonance imaging (MRI)] validated diagnosis of moderate to severe TBI with predominantly diffuse lesions made by the neurosurgeon (ISN); moderate and severe TBI diagnose: (1) coma >6 h after trauma or post-traumatic amnesia >24 h; (2) absence of intracranial lesions with significant ischemic or mass effect lesions (hematomas associated with TBI >25 cm^3^ or deviation from the midline >5 mm); and (3) Glasgow Coma Scale (GCS) <13 points at the hospital admission.

#### Exclusion Criteria

This study excluded patients with visible lesion on the left DLPFC detected by CT or MRI; current addictive behavior and/or severe psychiatric illness; uncontrolled epilepsy; current pregnancy; implanted metallic or electronic device carriers, such as a cardiac pacemaker, stents, epidural, or deep brain electrodes, cochlear implants, drug infusion systems or intracranial clips, and focal lesions.

### Randomization and Blinding

This exploratory *post-hoc* analysis of a double-blind, randomized control trial with parallel groups. After confirmation of eligibility and baseline assessment, patients were randomly allocated to Active or Sham rTMS conditions at a 1:1 ratio. Randomization was performed using a web-based tool (www.randomization.com) that generated a list of 4 block sizes. Patients were randomly assigned to the active or sham group using opaque envelopes that were sealed and numbered sequentially. The rTMS technician and the neuropsychologist (PAR) did not participate in the randomization, recruitment, or group assignment processes, however the rTMS technician was aware at the time of stimulation to which group the patient had been allocated.

### Intervention: High-Frequency Repetitive TMS (rTMS)

rTMS procedures were applied with a magnetic stimulator (MagPro X100, MagVenture A/S, Farum, Denmark) connected to a figure-of-eight coil. Two different coils were used: (1) Active coil (110 mm external diameter, C-100®, MagVenture Tonika Elektronic, Farum, Dinamarca) and (2) Sham coil (MC-P-B70®, Magventure Tonika Elektronic). The sham coil was identical to the active coil in shape, color, and noise emission. The stimulation intensity used in the active therapeutic condition was set to 110% of each subject's motor threshold, defined as the lowest intensity of the machine (measured as a percentage of its maximum power) capable of evoking a motor evoked potential (MEP) in at least five of 10 attempts (71).

RTMS was performed with the figure-of-eight coil disposed tangentially to the convexity of the head on the left dorsolateral prefrontal cortex (DLPFC). The target location on the scalp was identified on the 1st day of the rTMS protocol and was based on the International 10/20 electroencephalography EEG—system for aided by a tool developed by Beam et al. (72). Experimenters stimulated the F3 location, which represents the left DLPFC.

Trains of high frequency (10 Hz) rTMS were delivered in short periods (5 s duration) separated by longer periods of no-stimulus (25 s). A total of 2,000 pulses were applied at each daily session (40 stimuli/train, 50 trains), for 10 sessions.

### Safety Issues

This study involved the participation of the medical committee, whose members were directly involved in overseeing the TMS sessions. Committee members are tasked with un-blinding participants after adverse events, patient complaints, or at IRB request. Before the first rTMS session, all participants answered a standardized screening questionnaire with safety-related questions adapted from Rossi et al. (73). After each session, patients are given an adverse effects questionnaire. Any spontaneous complaints related to the stimulation were also recorded.

### Instruments

#### Screening

The severity of TBI was defined using the Glasgow Coma Scale (GCS) (74) collected at hospital admission. Participants with GCS<13 were included.

State-Trait Anxiety Inventory (STAI)—State (70), validated in Portuguese (69): participants with more than 42 points at STAI were included in the study, based on the cut-off criteria for the Brazilian population (69).

#### Assessment

State-Trait Anxiety Inventory (STAI)—State (75), validated in Portuguese (69): the STAI is a self-report scale in which patients are asked to rate how much they identify with of 20 statements related to anxiety based on a 4 point Likert scale. STAI-State evaluates a transient emotional state characterized by subjective feelings of tension that may vary in intensity according to context.

The Beck Depression Scale or Beck Depression Inventory, 2nd edition (BDI-II) (76, 77): a self-report questionnaire with 21 multiple-choice items. It is a widely used instrument for measuring the severity of depressive episodes.

##### Executive function index

Researchers calculated the index score using the average z scores of:

(a) Stroop Test Victoria Version (78): assesses inhibitory control by asking subjects to rapidly name colors distributed on cards with distracting color-words;
(b) Five Point Test (78): assesses the ability of visual fluency and flexibility by modifying visual patterns
(c) Backward Digit Span (79): evaluates working memory capacity by asking subjects to repeat a string of numbers in reverse order.

### Data Analysis

Psychiatric scores (STAI and BDI-II) were analyzed as raw data. The Executive Function Index were calculated as a *z*-score.

### Statistical Analysis

We estimated a sample size of 36 subjects based on previous TMS study (52). For this *post-hoc* analysis, all outcome measures were analyzed only in cases that completed the rTMS protocol. For comparison of the demographic data at baseline between groups (active vs. sham rTMS), we used *t*-tests or Kruskal Wallis tests for continuous variables (i.e., age, years of schooling, time port trauma) and a chi-square analysis for categorical variables (mechanism of trauma). Mixed-effect model (REML) regression analyses were used according to our research hypothesis. We considered each participant as a random effect, the group (active and sham), the time (baseline, first, and second assessment), and the interaction between the group and the time as fixed factors. The chi square test was used to rule out the significance of side effects. A significance level of *p* < 0.05 was used for all tests. The statistical analyses were performed using STATA IC16 software.

## Results

**Figure 1:**
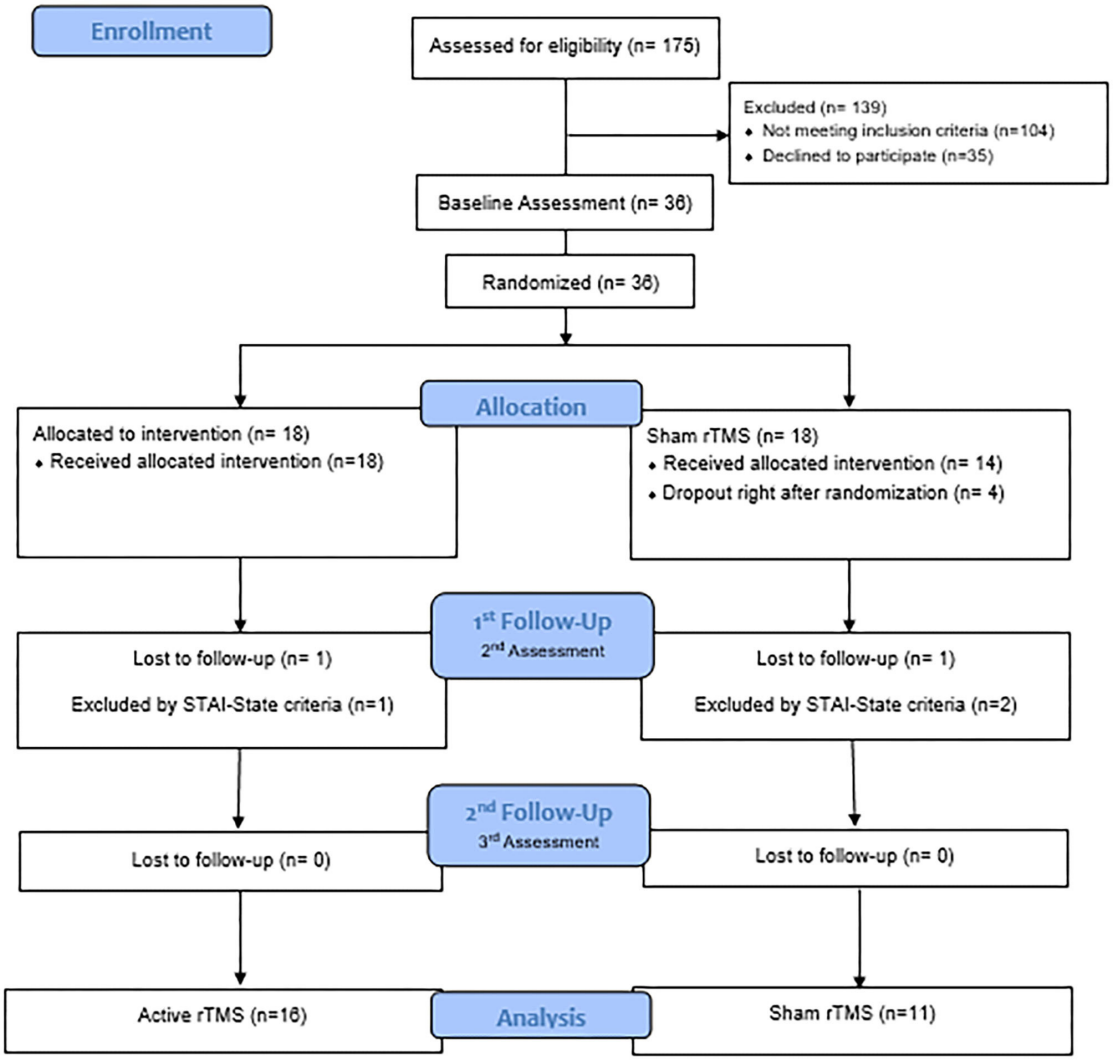
Flow diagram. CONSORT STATEMENT.

The flow diagram describes the participant selection process of this study (Figure 1). Thirty-six patients were initially recruited and the final remaining sample were 27 patients: *n* = 16 in the experimental intervention group and *n* = 11 in the sham group. Three participants were excluded because they did not meet the inclusion criteria for anxiety symptoms (*n* = 1 from the active group and *n* = 2 from the sham group). Four participants dropped-off immediately after the randomization (*n* = 4 from the sham group), and two lost the follow-up during the treatment (*n* = 1 from the active group and *n* = 1 from the sham group).

Demographic characteristics of the sample are reported in Table 1. No differences on demographic measures were observed between the groups (*p*_s_ > 0.05).

**Table 1:** Demographic characteristics of the study participants of the *post-hoc* analysis.

**Demographic characteristics**	**Active group *n* = 16**	**Sham group *n* = 11**	***p*-value**
Male—*n* (%)	14 (87.5)	10 (90.9)	
Age at DAI years—mean (SD) [range]	32.8 (13.3) (19–64)	31.6 (11.3) [18.0–49.0]	0.54^***^
Scholar—mean years (SD) [range]	10.1 (3.1) (4–16)	10.6 (1.8) [7.0–12.0]	0.65^****^
Time after the DAI—mean months (SD) [range]^*^	17.8 (3.7) [12.0–26.0]	17.6 (2.1) [15.0–22.0]	0.86^****^
**Causes of the DAI**			
Vehicle accident—*n* (%)	5 (31.2)	4 (36.4)	0.60
Motorcycle accident—*n* (%)	7 (43.7)	4 (36.4)	0.59
MVC pedestrian—*n* (%)	2 (12.5)	2 (18.2)	0.70
Fall—*n* (%)	1 (6.2)	0 (0)	
Physical attack—*n* (%)	1 (6.2)	1 (9.1)	0.79
GCS mean (range)^**^	4.0 (3.0–6.0)	3.0 (3.0–5.5)	
GOS-e mean (range)	6.0 (5.0–7.0)	6.0 (6.0–7.0)	

* *Missing values n = 1 (3.3%)*,

** *Missing values n = 2 (6.6%)*,

*** *Kruskal-Wallis*,

**** *T-Test*.

*DAI, Diffuse Axonal Injury; MVC, motor vehicle collision; GCS, Glasgow Coma Scale; GOS-e, Glasgow Outcome Scale-Extended; SD, Standard Deviation*.

The raw data of the results are displayed on Table 2.

**Table 2:** Descriptive results of the outcomes over in the baseline (time 1), after the last rTMS session (time 2), and at 3 months follow-up (time 3).

**Variable**	**Time**	**Active group**	**Sham group**	
		**M (SD) [range]**	**M (SD) [range]**	*p*-value^*^
STAI-state	1	56.5 (6.6) (44–65)	56.4 (8.1) (42–68)	
	2	56.6 (11.9) (36–77)	63.3 (11.8) (48–82)	0.15
	3	63.6 (14.7) (51–107)	58.5 (12.1) (38–73)	0.44
BDI-II	1	13.5 (10.5) (1–34)	11.7 (10.8) [0–35]	
	2	9.2 (8.6) [0–30]	7.8 (6.2) [0–19]	0.95
	3	8.4 (9.0) [0–32]	4.9 (5.5) [0–19]	0.54
EF index	1	1.3 (0.4) [2.1–0.7]	1.3 (0.4) [2.0–0.68]	
	2	1.1 (0.6) [2.15–0.4]	1.2 (0.6) [2.0–0.35]	0.72
	3	1.1 (0.6) [2.0–0.7]	1.0 (0.5) [2.1–0.4]	0.50

* *Statistical analysis of the interaction between group (active and sham) and time (1 vs. 3, and 1 vs. 3). M, Mean; SD, Standard Deviation; BDI-II, Beck Depression Scale or Beck Depression Inventory 2nd edition; EF index, Executive Function index; STAI, State-Trait Anxiety Inventory*.

**Figure 2:**
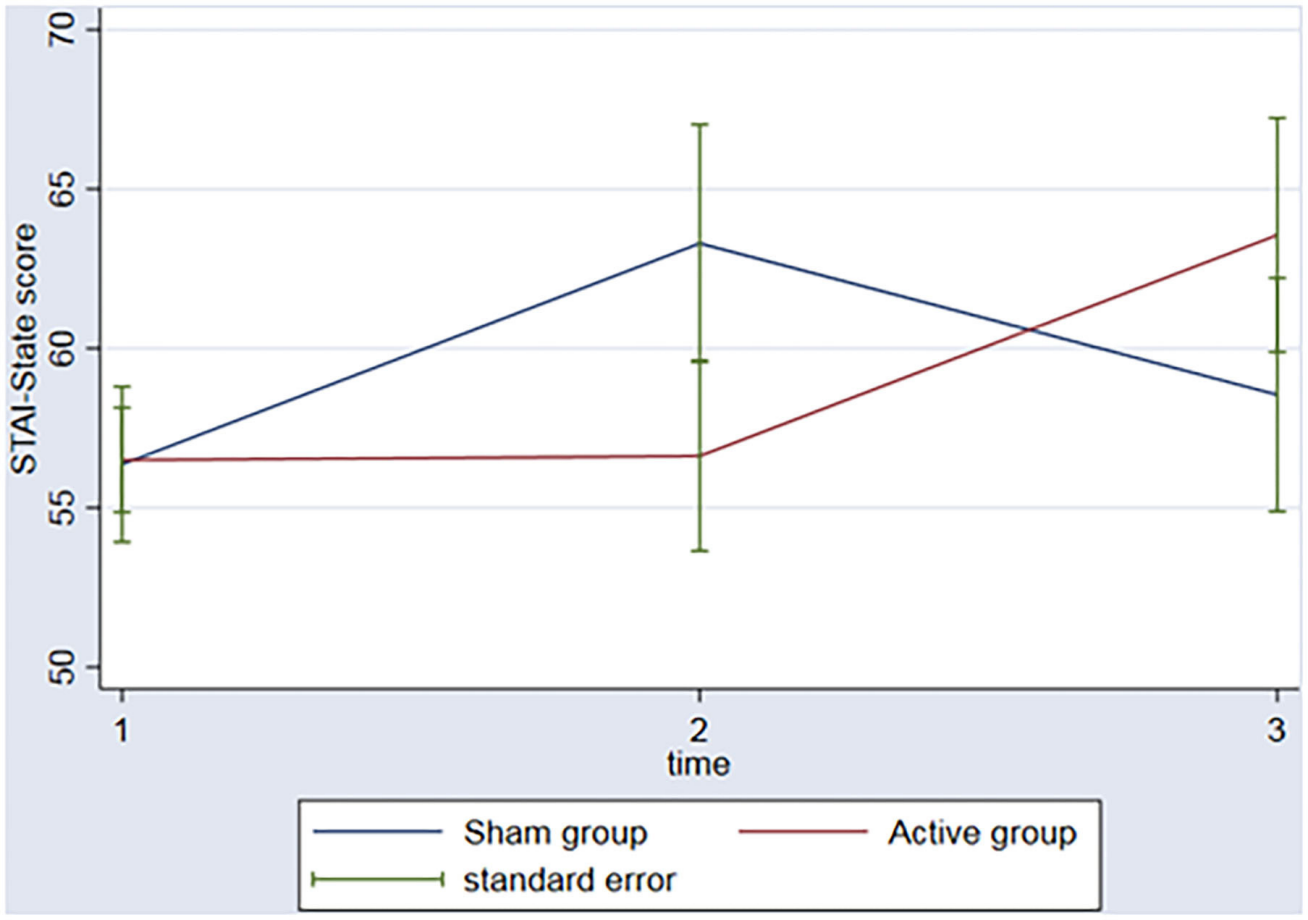
Graph: STAI-State scores during the 3 timeponts (baseline (1), after the rTMS (2), 3 months follow-up (3)) for both sham and active group.

The groups did not have significant changes in the STAI over the time (*p*_s_ > 0.06) nor interaction between group (active vs. sham rTMS) and time (baseline vs. 2nd assessment vs. 3rd assessment) (*p*_s_ > 0.14) (Figure 2).

**Figure 3:**
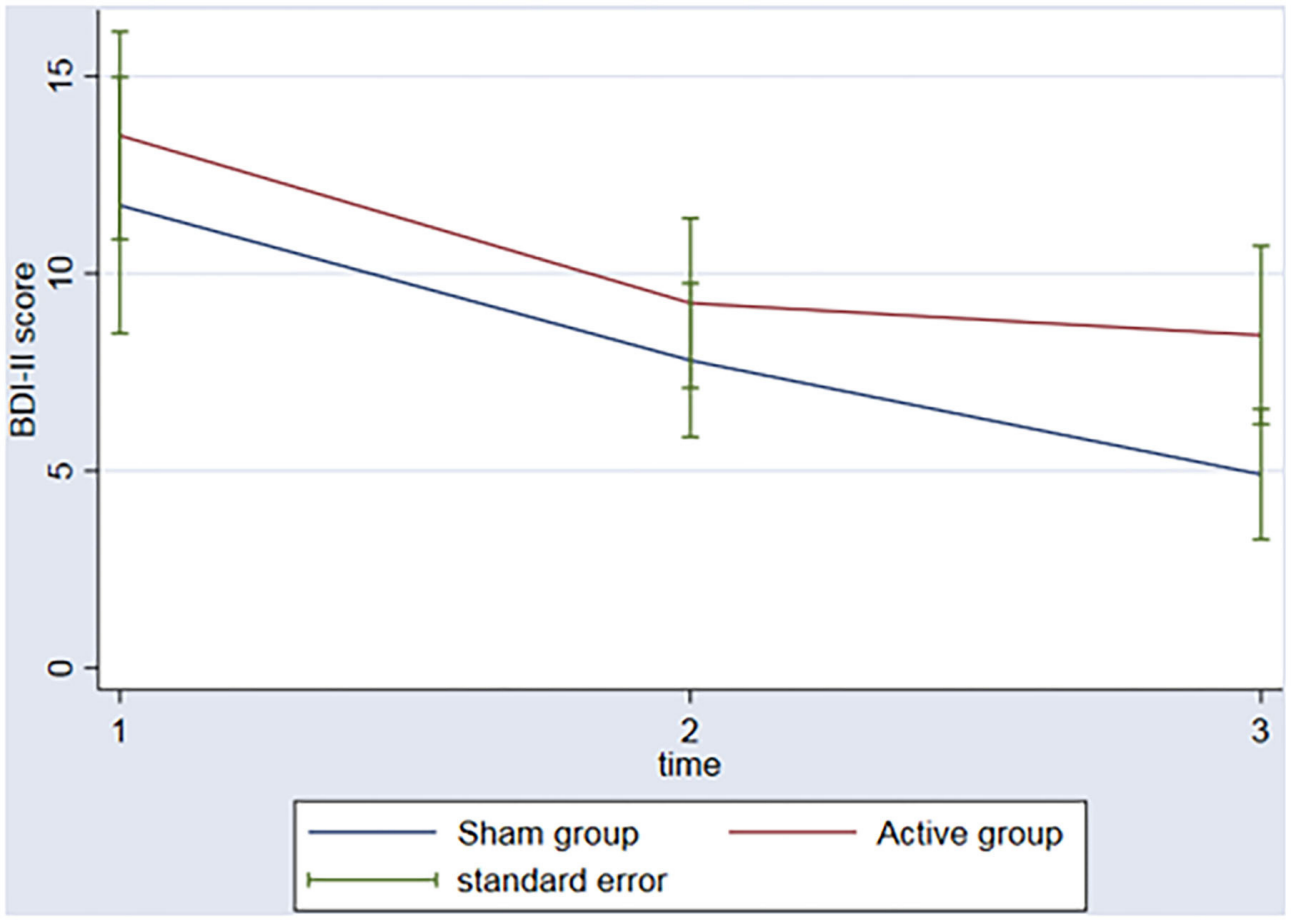
Graph: BDI II scores during the 3 timeponts (baseline (1), after the rTMS (2), 3 months follow-up (3)) for both sham and active group.

**Figure 4:**
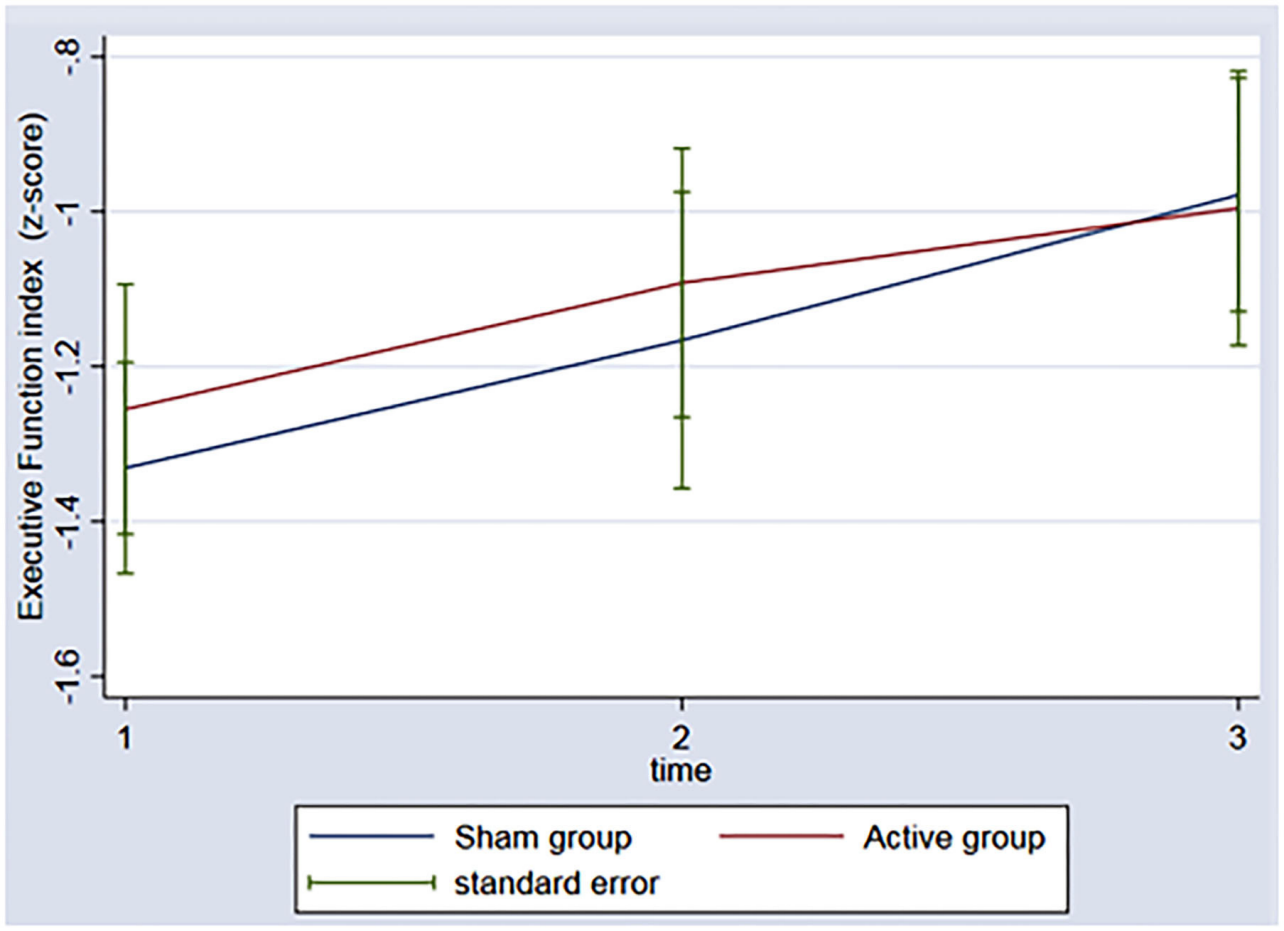
Graph: Executive function index during the 3 timeponts (baseline (1), after the rTMS (2), 3 months follow-up (3)) for both sham and active group.

In regard to the depressive outcome, there was a significant main effect of time on depressive scores on the BDI-II (Figure 3, *p* = 0.002) between baseline and the 3rd assessment. There was no interaction between time and group (*p* = 0.53). An evaluation of executive function over time was also conducted. The REML analysis shows an increase in executive function index scores over time (*p* = 0.001) between baseline and the 3rd assessment; however, no interaction was found between group and time (*p* = 0.50) (Figure 4).

No significant adverse effects were reported between the groups after the first (*p* = 0.23) and the second week of stimulation (*p* = 0.29).

## Discussion

This is the first study *post-hoc* analysis investigating the efficacy of 10 sessions of rTMS on anxiety in participants with moderate to severe chronic TBI. Contrary to our hypothesis, results show that high frequency rTMS had no significant effect on improving anxiety symptoms in this population. However, an improvement in depressive symptoms and executive functions occurred in both the active and sham groups over time. There are two main hypotheses regarding the lack of significant change in anxiety symptoms after rTMS. The first is relates to the characteristics of a lesioned brain, which differs biologically and pathologically from an intact, non-injured brain, in a way that the structural characteristics of the brain seem to be key to understanding the distinct effect of the rTMS in persons with TBI and persons with psychiatric disorders. Additionally, the nature of the diffuse lesions resulting from moderate to severe TBI is very complex and differs from focal lesions, which may have contributed to the negative result.

The second hypothesis is related to the frequency of the rTMS. High frequency rTMS over the DLPFC has been shown to improve clinical outcomes such as depression and anxiety in patients with neurological diseases, such as Parkinson's disease (63). On the other hand, low frequency rTMS over the right DLPFC has also been successfully used to treat anxiety symptoms in persons diagnosed with Generalized Anxiety Disorder (44, 45), Panic Disorder (53) and Post Traumatic Stress Disorder (PTSD) (56, 57, 80). For this study, we selected high-frequency rTMS following methods of these preceding studies (81).

In psychiatric patients without brain lesions, inadequate top-down inhibition of the amygdala by the anterior cingulate cortex or medial prefrontal cortex (PFC) is associated with anxiety (82, 83). Alterations to the anterior cingulate cortex (medial PFC), hippocampus, and amygdala post-TBI result in symptoms that resemble PTSD or other anxiety disorders (84). Animal models of diffuse traumatic brain injury identified alterations in limbic circuitry including increased neuronal hyperexcitability and GABA production proteins (84, 85). The limbic structures are generally more vulnerable to TBI (86), resulting in metabolic, electrochemical and inflammatory perturbations (87), leading to clinical manifestations that include sustained anxiety symptoms (88). Because there are components of dysregulation in both inhibitory (89, 90) and excitatory pathways (91–93) after brain injury, both high and low frequency rTMS may reach the damaged neurocircuitry, improving anxiety symptoms in this population.

In regards to our secondary outcome, the improvement in depressive symptoms for both groups (active and sham) over time may be attributed to a placebo effect of the intervention. Studies have shown that the placebo effect can occur in different intervention modalities, including NIBS (94–97). Patients with sustained cognitive deficits due to TBI who have not undergone neurorehabilitation treatments may be more susceptible to the placebo effect than healthy controls (19, 20). One possible explanation is that, for many patients, the study protocol is the first rehabilitative care received since injury, which inflates patient expectations and commitment levels during the research activities (98). In this study, participants are evaluated over 13 sessions, including the rTMS and assessment visits, and patients generally develop a strong rapport with hospital staff. Such positive social interactions are known to elicit feelings of care and well-being, favoring the improvement of the patient's response to treatment (20). In clinical trials of antidepressant medications, positive placebo effect appears to be correlated with functional changes in the ventromedial prefrontal structures and the posterior midline and striatal regions, ultimately promoting changes in cognitive processes related to self-assessment and self-perception skills (99). Similarly, the DLPFC region may be related to maintaining and updating expectations that drive the placebo effect by modulating cortical pathways (94). In persons with TBI, the placebo effect has been associated with an increase in neurotransmitter activity and improvement the patient's commitment to the rehabilitation process (100).

This study *post-hoc* also found improvements in executive functions of both sham and active groups over time. Many studies have associated depressive symptom scores with performance in executive tasks (101, 102). In our sample, the improvement of depressive scores followed improvement on the executive function index. However, further analysis needs to be done to confirm this hypothesis.

Although the literature points to the positive effects of rTMS on other neurological and psychiatric pathologies, moderate to severe TBI has unique pathophysiology, which may explain the lack of treatment response. Because of the focality of rTMS, a different modality NIBS may be more appropriate for this population. The fact that we recruited patients in the chronic phase may also be a limitation of this study. The induced neuroplastic effects of rTMS may be more significant in the acute and subacute stages of TBI. However, patients in the chronic phase are at a lower risk for epileptic seizures and are subsequently a safer cohort to put through new rTMS protocols. Another potential limitation is the small sample size. The loss of follow-ups may have underpowered the results. In this way, we encourage further studies to replicate our findings and verify the potential effectiveness for the high-frequency rTMS to improve psychiatric symptoms in persons with moderate to severe closed TBI. Finally, rTMS as a stand-alone intervention for anxiety may be less impactful than a treatment plan that combines rTMS with cognitive behavioral therapy and/or pharmacological therapy (103).

In conclusion, in this *post-hoc* analysis, high frequency rTMS did not improve anxiety symptoms in patients with moderate to severe TBI as compared to a sham group. Still, limitations to these results might be considered.

## Data Availability Statement

The raw data supporting the conclusions of this article will be made available by the authors, without undue reservation.

## Ethics Statement

The studies involving human participants were reviewed and approved by Research Ethics Committee (CAPPESQ) of HCFMUSP under n°. 1.151.170. The patients/participants provided their written informed consent to participate in this study.

## Disclosure

Financial Disclosures for the previous 12 months: All funding sources, not related to the current research, for each author: AZ: The Collaborative Center for X-Linked Dystonia Parkinsonism and NIDCD 3R01DC017291-02S1. WP: reports grants and non-financial support from National Institute for Health Research (NIHR), during the conduct of the study.

## Author Contributions

PR collected neuropsychological data and wrote the manuscript. AZ did the statistical analysis and wrote the manuscript. HV, AB, and MT revised the manuscript. IN contributed to the recruitment of patients, collected data, and revised the manuscript. CH made the data collection and revised the manuscript. VG contributed to the recruitment of patients and revised the manuscript. WP contributed to the development of the project design and revised the manuscript. All authors contributed to the article and approved the submitted version.

## Conflict of Interest

The authors declare that the research was conducted in the absence of any commercial or financial relationships that could be construed as a potential conflict of interest.
